# Hydrochorous Seed Transport in the Lower Traisen River before and after Riverbed Restoration

**DOI:** 10.3390/plants12132409

**Published:** 2023-06-22

**Authors:** Leonid Rasran, Kati Vogt, Marc Trattnig, Karl-Georg Bernhardt

**Affiliations:** Institute of Botany, Department of Integrative Biology and Biodiversity Research, University of Natural Resources and Life Sciences (BOKU), Gregor-Mendel-Straße 33, A-1180 Vienna, Austria

**Keywords:** dispersal, monitoring, seed viability, invasive species, riparian vegetation, plant strategies

## Abstract

Hydrological restoration was carried out in a Lower Traisen, a small river within the floodplain of the Danube. The main goal was the reestablishment of typical riparian plant communities by using the potential of natural dispersal processes. We studied the transport of plant diaspores in the river water before and after the reconstruction of the riverbed. Aquatic seed traps were placed upstream and downstream of the restoration site. We identified the transported species and tested the viability of propagules. Functional species traits were analyzed to predict the probability of successful hydrochorous dispersal and changes in the transport pool due to the restoration. One-third of the local species pool was detected as being diaspores in the river. We observed a significant increase of ruderal species and neophytes, while the competitors and stress-tolerant competitors declined. Hydrochory is an important dispersal pathway for numerous plant species in the study area, including those without specific adaptations to this vector. Hydrochorous transport appears to be a sink for large-seeded species, primarily adapted to endozoochory. Follow-up management should be recommended to control the invasive species and to improve the structural and biological diversity of the Traisen Valley by supporting target species, which are also represented in the transport pool.

## 1. Introduction

In Central Europe, where nearly all the main rivers were heavily modified by channeling or damming, the favored method of restoring a riparian ecosystem is to reestablish the natural hydrological dynamic and the lateral connectivity between the floodplain and the river [1,2,3,4]. This goal can be reached, e.g., by removing water control structures, constructing artificial barriers and meanders, prolonging the riverbed, discharging water into the floodplain, and establishing new connections to oxbows and dry channels [5]. For the second main part of the restoration, the reestablishment of the riparian biota, the river itself as a corridor in the landscape, is assumed to be a crucial factor. It is commonly assumed that the restoration of abiotic conditions is sufficient and that the floodplain target species will appear through natural dispersal [6]. Unfortunately, this last point should be seen critically in the landscape, strongly affected by previous fragmentation and canalization of riverbeds [7,8,9]. For example, the success of restoration measures is dependent on whether the diaspores reaching the newly constructed sites belong to the natural riparian/floodplain vegetation (target species) or the transport pool is strongly dominated by ruderals and/or invasive species.

The dispersal of plant propagules with running water is an important, and for many species of riparian wetlands, it is a predominant way to spread their diaspores (see, e.g., [10,11,12,13,14]). The species pool of water-transported propagules, in some cases, even appears to be larger than that of the anemochorous seed rain [15,16]. That is why the knowledge about the potential of this dispersal pathway is important in the context of floodplain restoration, where hydrochorous dispersal is supposed to play a significant role (see, e.g., [9,11,17,18,19]). Hydrochory is a common process that affects many populations within riparian plant communities, including species with no specific adaptations to this dispersal vector. However, species’ composition of the dispersal pool is dependent on numerous additional factors, including the composition of the surrounding vegetation; in addition, specific adaptations of involved plants are expected to be meaningful [20]. The entire dispersal process can be seen as an ecological filter, with significant values of seed mortality within single steps, such as drift in the running water, deposition in drift lines, and seedling establishment [21,22]. In these steps, species that are occasionally flushed into the river are especially filtered out, while the successful dispersal, the ability to pass through the ecological filter, is often dependent on functional species traits. We assume, among other things, that life form and strategy [7,11,23], seed mass [24], seed release height [25], preference of maternal plants for vegetation communities typical for riverbanks and riparian landscapes in general [9,26], and adaptations to further dispersal vectors (wind, animals, and birds; see also [27]) are suitable predictors for successful seed transport with the stream. In reverse, changes of the functional traits of the transported pool can be—even better than the changes in species composition itself—sensitive indicators for the significance of spatial and temporal transformations of the riparian landscape in the course of restoration. In the following project, we compared the transport pool of a small river in the Danube floodplains before and after hydrological restoration, focusing on the following questions:

i. How does the transport pool in running water change due to hydrological measures?
ii. Could functional species traits be used as predictors for the survival of transported diaspores and for special/temporal changes?

For this purpose, we sampled the surface of the river Traisen upstream and downstream the main restoration area at two different time periods—shortly after the start of the project in 2014 and after the end of riverbed reconstruction in 2017. We focused on the diversity of the transported species and on the functional species traits that are relevant for hydrochory and survival of the diaspores.

## 2. Results

At the study area, Traisen, in 2014, we recorded about 170 taxa (9500 individuals) as seeds and 80 taxa (2200 individuals) as seedlings within five weeks. The most frequent seeds were *Alnus glutinosa* (L.) Gaertn. (34.3%), *Urtica dioica* L. (18.7%), and *Betula pendula* Roth (12.7%). The most frequent taxa in the seedling analyses were *Urtica dioica* (23.8%), *Alnus glutinosa* (21.4%), *Cyperus fuscus* L. (11.1%), and *Humulus lupulus* L. (7.2%).

The regional species pool of the community of Zwentendorf (53.9 km^2^ around the study area), included approx. 1000 taxa of vascular plants, from which about 300 taxa (local species pool) occur close to the riverbank and within the floodplain area affected by restoration measures (Bernhardt, unpublished). Within the sampling period of five weeks, we found 39% of the local species pool to be represented as seeds in the river water. A few species were transported in the river but were absent in the local vegetation of the riverbank; these were mainly arable weeds and crop/garden plants. They made up not more than 7% of the transported species.

### 2.1. Trait-Dependent Differences between Total and Viable Seed Transport (2014)

The samples of the first study period (2014) showed significant differences between the two methods applied (seeds vs. seedlings). The differentiation of clusters based on functional traits (Table 1) had even higher explained variation than clustering based on species composition (Table 1). Nearly all regarded traits that were tested separately showed highly significant differences in their community-weighted means (CWMs) between the samples of seeds and seedlings. However, global permutation tests based on axis scores and community-weighted trait means of all analyzed traits together were not significant (pseudo-F = 0.7, *p* = 0.761; Table 1). Thus, none of the CWMs was a significant predictor for the differences between seeds and seedlings. Nevertheless, we continued the interactive forward selection analysis to distinguish traits with the highest (potential) explanatory value. Such traits appeared to be the dispersal mode endozoochory and competitive/stress tolerant strategy. Both traits were negatively correlated with the survival of species during the transport (Table 1), and their explanatory value was about 7% each.

### 2.2. Composition of Viable Seeds in 2017

In the ten weeks of the second sampling period, considering only viable seeds (seedlings), we detected about 26,750 individuals belonging to 100 taxa (1/3 of the local species pool; 1/10 of the regional species pool). The most common species was again *Urtica dioica* (36.4%), but the second most common taxa was *Solidago gigantea/canadensis* (28.2%), while *Alnus glutinosa* and *Cyperus fuscus* were less common (0.2% and 1.3%, respectively, of all seedlings). The vast majority of *Urtica* seeds (9500 of 9750) was captured at the upstream Bridge (B), while for *Solidago*, the downstream bridge was the main location (with 7200 out of 7550 seeds).

**Figure 1:**
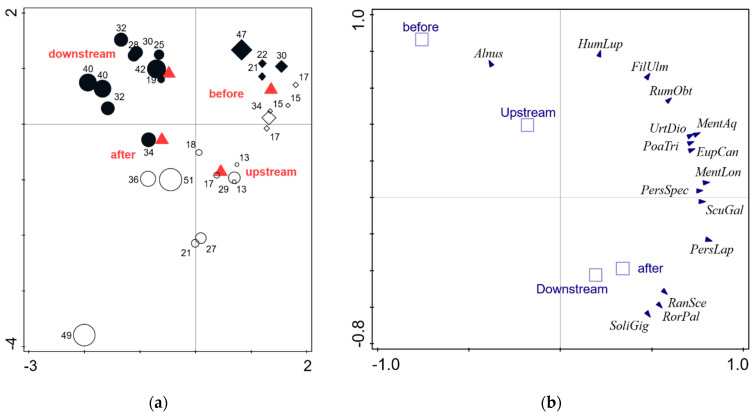
Results of the multidimensional analyses. (**a**) Redundancy analysis (RDA) ordination, the relationship between the sampling time and sampling according to the species composition of transported diaspores. Numbers indicate the number of species per week. Explained variation for Axis 1—19.3%; cumulative for Axes 1 and 2—22.2%; pseudo-F = 3.7, *p* = 0.002. (**b**) PCA with supplementary variables. Position of the most common species in relation to the sampling time (2014—diamonds vs. 2017—circles) and sampling position (upstream—open symbols vs. downstream—filled symbols). Explained variation for Axis 1—32.2%; cumulative for Axes 1 and 2—49.7%.

Consequently, the species composition differs significantly between sampling times and locations, even though the entire explanatory power of these two parameters was not very high. Samples from upstream and downstream and from 2014 and 2017 build separate clusters (Figure 1).

The analyses of community-weighted means of some species traits (including strategies and affinity to different plant communities) showed significant differences for many of these parameters between the sampling periods and, in some cases, also between upstream and downstream sites (Table 2). After the hydrological measures, we observed a general increase in the number of small seeds, seeds of annual species, ruderal competitors, and, in general, species of ruderal plant communities. In contrast, the transport of stress-tolerant competitors, tree seeds, including species of alder carrs, declined. Among others, we observed an increase in the number of diaspores of perennial ruderals between the upstream and the downstream site. The autochthonous species *Urtica dioica* and the neophytic species *Solidago* (*S. gigantea*/*S. canadensis*) were counted in this category. A significant increase of *Solidago* cover could be observed in the study area between both sampling periods, and this is provable by the ANOVA comparing the effect of neophytes (Table 2). The numbers of diaspores of some stabile plant communities (perennial grasslands and reeds) were less affected by temporal or spatial effects.

Regarding seed buoyancy, we registered a significant interaction between the spatial and temporal effects. In 2014, there were generally more species with high buoyancy, especially in the downstream samples. Later on, we mainly found less buoyant species, and the CWM of the floating time of species in the upstream samples was higher than it was in the downstream samples. There was also an increase in epizoochoric and myrmecochoric species in the downstream samples after the hydrological restoration; however, in 2014, the contribution of these species was generally lower and more significant in upstream samples.

## 3. Discussion

The sampling period of five-to-ten weeks was comparatively short, and with the three traps of 16 cm in diameter, only about 1-2 percent of the surface of the river Traisen (which is about 50 m broad at upstream sampling site and between 20 and 25 m broad at downstream sampling sites) could be sampled. Nevertheless, a significant part of the local and even regional species pool was registered as diaspores in the river water, confirming the general importance of this pathway for the biodiversity of the Traisen floodplain. The sampling period within the late summer and autumn was chosen according to the experiences we had with our previous studies [27,28]. At this time, the hydrochorous seed transport reaches high values due to the phenology of riparian species (the majority of them produced ripe seeds, which were released mainly in early autumn) and weather conditions, such as heavy rainfalls, flushing high amounts of the previously released seeds into the river [14,29]. We observed at least one such event during both sampling periods. The exchange of the downstream locality was indispensable, as the sampling site of 2014 (Bridge A) was cut off from the main river during the course of the restoration and turned into an oxbow. However, the water flow quantity remains the same, and the changes of the channel were direct results of the restoration measures, allowing for the before/after comparison. The distance between the upstream and downstream locations increased from approx. 4.5 km in 2014 to 7.2 km in 2017. The main consequence should be seen in an increased connectivity between the river and its surroundings and the greater possibility for diaspores to enter the Traisen River. For the seeds already floating on the water’s surface, an additional hour of the flotation would not be expected to make much difference, as the buoyancy of most of the species is numbered by days or weeks.

In spite of the abovementioned diversity of species and functional groups in the transport pool, we noticed that only a number part of regionally occurring plants profit from the dispersal pathway of the river water. Quite a few species strongly dominate the transport, while the majority of further taxa is represented by a very small number of propagules. This disproportion is already well-known from the studies on hydrochory [8,9,28], as well as for other dispersal vectors, in general [30,31]. The transport itself is an additional ecological filter, reducing the viability of diaspores, which are less adapted for this method of dispersal. For instance, endozoochorous species, especially avichorous ones with fleshy fruits (*Prunus* spp., *Rubus* spp., and *Sambucus nigra*), nearly never germinate after the drift. Most of these fruits are expected to fall into the river directly from the maternal plants or be washed in with rain or snowmelt without digestion. We assume that endozoochorous seeds need this special prerequisite (digestion), and the absence of this step led to a low viability or germinability after water transport (compare with [32]). Each parameter (dispersal mode endozoochory and competitive/stress tolerant strategy) explains only about 7% of the differences between generally transported and viable (surviving) diaspores and are negatively correlated with survival. A similar analysis performed on the data of the seed transport and germination pool of the Upper Eider River (Northern Germany) also showed an explanatory value of less than 20% for functional traits/strategies of the plants [27]. In the case of the Upper Eider River, the explanatory traits were represented by R/SR-Strategy (ruderals and stress-tolerant ruderals) and the dispersal mode anemochory/dust (both positively correlated with survival). Moreover, dust flyers are usually small compact seeds that are less vulnerable for aquatic organisms compared with endozoochorous ones (see, e.g., [33]). The seeds of terrestrial endozoochorous species can be accidentally consumed by fish and are thus lost for further transport [34]. The differences in anatomy/mechanical resistance of the seed coat between the species could be one further explanatory parameter which is currently not considered adequately. This could be an important topic for future studies.

At the time that the Traisen was straightened and directly flowing, the diaspores of species with high floating ability were overrepresented. A change in this tendency after the hydrological restoration can be seen as a sign of the increased connectivity of the floodplain, where also species with a short floating time of the seeds contribute to the transport pool within the local area, while buoyant seeds arriving at the upstream sample site can be interpreted as dispersed over (potentially) longer distances (compare to [35]). A similar explanation can be used for the increase of epizoochorous and myrmecochorous species. These two dispersal vectors normally demand specific adaptations, contradicting the floating ability or ability for hydrochorous dispersal in general. Their presence in the transport pool is evidence for the generally high potential of the Traisen River as a dispersal pathway, a typical case of “chance dispersal” sensu Berg [36]. This phenomenon is typically observed at small spatial scales, but it can also play an important role for long-distance dispersal [37].

The consequences of the hydrological measures in the Lower Traisen were changes in the landscape’s structure, especially the reduction of the alluvial forest area and creation and enlargement of sandbanks and mud banks along the river. These changes were clearly reflected by the decrease of seeds of woody species and the increase in the number of pioneers and ruderals.

The general statement that strong competitors (C-strategy plants) are less likely to be transported and establish after hydrochorous transport than ruderals (R-strategy plants; see, e.g., [7]) was tendentially enforced by the comparison of the unrestored (2014, upstream) and hydrologically restored situation (downstream, 2017). Perennial ruderals are additionally supported by the high nutrient status of the alluvial soils and are generally characterized by a high seed viability and germinability. Their dominance can be significant, especially for a short time after a large-scale disturbance [38].

The Traisen area is strongly affected by invasive plants (*Solidago gigantea*, *Impatiens glandulifera*, and *Ailanthus altissima*), which are well represented among drifted seeds and also seedlings. Especially for *Solidago*, a significant increase could be observed at the sites close to the new riverbed, which became more open due to restoration measures. This increase is clearly detectable in the hydrochorous transport. For invasive species in Central Europe, water dispersal is an important vector, and many of them are considered to be river corridor plants [39,40,41]. For the study area of the Traisen River, additional management with the goal of the reduction of invasive neophytes is also carried out in course of the restoration project [42]. This should help to shorten the phase of the dominance of the ruderal species and to establish a closed alluvial vegetation (meadows and riparian forests) that is less vulnerable to biological invaders.

## 4. Materials and Methods

### 4.1. Study Area—Lower Traisen River (Danube Floodplain, Lower Austria; 48°22′ N, 15°49′ E)

**Figure 2:**
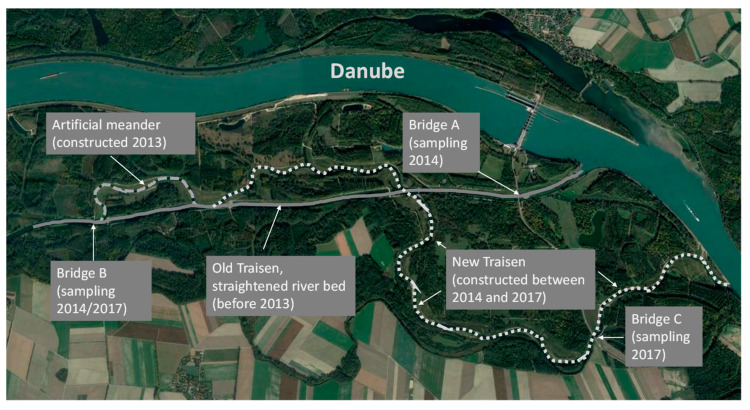
Study area, Lower Traisen, with river restoration measures and sampling sites.

The Lower Traisen is one of the Danube tributaries in Lower Austria, approx. 40 km upstream of Vienna. Its catchment area of approx. 1000 km^2^ can be divided into mountain and lowland parts. In the latter case, the Traisen flows about 30 km long through urban (St. Pölten) and agricultural landscapes (arable fields and vineyards), as well as through some floodplain forests and meadows of the Danube Valley. This part of the river has been strongly straightened and regulated since the beginning of the 20th century. Since 2013, restoration measures (Life+ Traisen project) have started in the lower section of the river course (within the Danube floodplain, shortly before entering the Danube; Figure 2). These restoration measures aim to increase the natural dynamics of the river and lead, among other things, to the development of pioneer communities of riverbanks and riparian willow scrubs. In its lower part, the riverbed is between 25 and 40 m broad, with an average discharge of 14 m^3^/s, but this value can increase more than tenfold during flooding [43]. Floods mainly occur in spring, but they can also take place during other times of the year (late summer/autumn) in the case of strong rainfalls. All study locations belong to the market municipality Zwentendorf in the state of Lower Austria.

### 4.2. Sampling

The sampling of drifted seeds in the Lower Traisen, the part of the river that is most affected by the restoration measures, occurred during two time periods. The first is the sampling period that covered 5 weeks between 14 October and 18 November 2014. At that time, the first artificial meander was already created (Figure 1), but the main flow occurred via the old straightened part of the river. The downstream sampling site was the bridge close to the hydroelectric powerplant Altenwörth (Bridge A). The upstream sample site (Bridge B) is situated north of the village of Preuwitz. The second sampling period took place between the end of August and October 2017 and lasted 10 weeks. At that time, the main restoration measures were finished, and the riverbed of the old straightened Traisen was no longer active. Thus, as the downstream location, another bridge on the road between Bärndorf and Altenwörth (Bridge C, approximately 1.5 km south of Bridge A) was chosen. The upstream location was the same as in 2014 (Figure 2).

We sampled the river surface by using aquatic seed traps (see [28] for a detailed description). Each of the two traps was exposed per bridge and week.

For the sampling period 2014, the material caught by each trap was divided into two equal subsamples. One subsample was dried and manually searched for plant diaspores, using a stereo lens. All generative propagules were counted and identified using a reference seed collection and the literature (especially [44]). The other half of the sampled material was freshly exposed in a layer of less than 1 cm on sterilized soil (commercial garden soil: TKS 2, Floragard TM) in an experimental garden at the University of Natural Resources, Vienna, for germination. To prevent external seed input, the samples were put in a so-called “grid house” (a lattice house with a glass roof), where they were exposed to natural temperature conditions for a period of at least one year in order to break seed dormancy and watered regularly to prevent desiccation. All seedlings were determined, counted, and removed. In some cases, if the seedlings could not be determined up to the species level, they were transferred to a greenhouse for later determination. In this way, both the potentially transported species (seeds) and those that remained viable during and after the transport and were able to germinate under the typical conditions of the riverbank (seedlings) were registered.

For the sampling period 2017, the entire trapped material was exposed for germination in the same way as in 2014, and only seedlings were registered.

### 4.3. Species Traits

For all taxa, including both those detected as seeds and seedlings in the trapped material (transport pool), we compiled a dataset including functional species traits. To complete this dataset, we used the databases D^3^ [45], Floraweb [46], BiolFlor [46], TRY [47], and SID [48] and further publications [13,29,49,50,51,52,53,54,55,56], as well as our own measurements (see Supplementary Materials for more details). To assign plant species to habitat types, we regarded their properties as characteristic species for plant communities. All regarded species were related to the eco-sociological groups according to [57], BiolFlor [46], and our own experience. We also marked invasive and crop species for the study region.

### 4.4. Data Analyses

Constrained ordination (redundancy analysis RDA) was applied to analyze the difference between dried/visually analyzed and germinated samples based both on species composition and CWM. Based on the axis scores of the ordination, an additional RDA with interactive forward selection of traits was performed to find out which of the regarded functional traits possess the highest explanatory value.

For the seedlings data of 2014 and 2017, we also used RDA to analyze and visualize the effects of “location” (upstream vs. downstream) and “time” (before and after the restoration measures) on the species composition of samples. Additionally, we performed two-way ANOVAs to compare the community-weighted means of single functional traits between the both locations and time periods.

The multivariate analyses were performed with CANOCO 5 [58], while the ANOVAs were carried out with R 4.2.2 [59].

## 5. Conclusions

A significant part of the local species pool profits from the riparian transport and the increase of connectivity within the river valley after the hydrological restoration. Ruderals (Rs) and ruderal competitors (CRs) are especially among the profiters, while competitors (Cs) and stress-tolerant competitors (CSs) decline. Hydrochorous transport appears to be a sink for large-seeded species that are primarily adapted to endozoochory. Viable seeds of invasive species are overrepresented in the transport pool, and their amount increased strongly after the hydrological restoration, while only a few species of alluvial meadows (target species) could be detected. Follow-up management (among other things, the mowing of alluvial meadows and part afforestation) is mandatory to control the invasive species and to improve the structural and biological diversity of the Traisen Valley, and the additional transfer of the target species that were absent or insufficiently represented in the transport pool was a significant part of this management [60]. This should be taken into consideration for future projects.

## Figures and Tables

**Table 1:** Comparison between the entire (seeds) and viable (seedlings) transport pool in 2014. Results 1 of the multivariate statistical analyses (RDA). CWM—community-weighted means of functional traits.

Partial RDA (Results of Permutation Test)	Axis	Eigenvalue	Explained Variation	*Pseudo-F*	*p*
**Ordination following species composition**					
Explanatory variable, method (seeds vs. seedlings); co-variables, bridge and week	1	0.1655	20.26%	4.3	0.001
**Ordination following CWMs of functional traits**					
Explanatory variable, method (seeds vs. seedlings); co-variables, bridge and week	1	0.346	37.85%	10.4	0.002
**Ordination following Axis 1 scores out of previous ordination (CWM), Interactive-forward-selection**	1	0.1409	14.09%	0.7	0.761
**Relevant traits**					
Dispersal mode: endozoochory			7%	2.8	0.092
CS-Strategy			7.10%	3.1	0.099

**Table 2:** Results of comparisons (two-way ANOVAs) of community-weighted means of different functional species traits between the time periods (2014 vs. 2017) and between sample sites (upstream vs. downstream bridge). F-values are given. Significant effects are shown in bold; * *p* < 0.05, ** *p* < 0.01, and *** *p* < 0.001.

Functional Trait	Time	Location	Interaction
Seedweight	25.06 ***	1.52	0.72
Seed size_length	11.11 **	2.33	0.99
Seed size_width	16.56 ***	4.4 *	3.66
Seed size_thickness	1.1	0.63	0.31
Reprod. strategy_only by seed	6.79 *	3.63	3.02
Reprod. strategy_mainly by seed	27.97 ***	0.19	0.02
Reprod. strategy_seeds and vegetative	42.12 ***	0.91	1.17
Reprod. strategy_mainly vegetative	0.25	0.54	2.59
Mid_height	34.24 ***	0.24	0.65
Buoyancy_day	19.13 ***	1.09	4.68 *
Dispersal_wind_hairs	10.2 **	1.67	2.97
Dispersal_wind_wings	11.11 **	1.00	1.92
Dispersal_wind_dust	11.24 **	15.67 ***	8.3 **
Dispersal_hydrochory	3.38	4.85 *	0.01
Disp_myrmechory	7.77 **	12.18 **	7.1 *
Disp_epizoochory	5.92 *	40.95 ***	26.6 ***
Disp_endozoochory	0.37	1.84	1.61
Coniferous and deciduous forests outside the floodplain (LNW)	0.08	37.59 ***	2.83
Floodplain forests and alder carrs (BAW)	41.15 ***	13.19 **	8.59 **
Pioneer vegetation of mudbanks (BI)	0.05	0.14	1.4
Aquatic macrophytes (H)	2.57	0.01	0.76
Agricultural grasslands (MA)	0.25	0.01	1.93
Wet meadows (MO)	0.77	3.77	0.34
Reeds and tall herb fen communities (PH)	0.36	1.98	1.71
Communities of perennial ruderals and edges (AR)	15.46 ***	6.35 *	3.14
Small sedge communities of nutrient-poor mires (SC)	0.43	0.9	0.81
Annual ruderals and arable weeds (SO)	0.04	1.89	0.64
Life span_polycarpic perennials	8.69 **	15.09 ***	16.61 ***
Life span_monocarpic perennials	0.07	1.04	1.44
Life span_annuals	5.99 *	2.89	0.71
Phanerophytes	55.57 ***	0.65	0.24
Hemicryptophytes	14.38 ***	6.93 *	9.69 **
Kryptophytes	9.67 **	18.59 ***	5.71 *
Therophytes	0.08	0.47	1.77
Strategy Grime_C	3.84	32.44 ***	18.67 ***
Strategy Grime_CS_S	23.14 ***	0.55	0.23
Strategy Grime_CR	4.97 *	2.36	0.64
Strategy Grime_CSR	0.13	0.9	0.01
Strategy Grime_R_SR	2.13	2.02	0.24
Ellenberg_moisture	8.58 **	25.84 ***	4.89 *
Ellenberg_nitrogen	24.83 ***	6.05 *	2.23
Ellenberg_light	8.58 **	25.84 ***	4.89 *
Neophyte	6.9 *	11.83 **	4.46 *
Invasive neophyte	7.49 *	12.03 **	6.61 *

## Data Availability

The data presented in this study are available as Supplementary Material (see above). Further details can be obtained on request from the corresponding author.

## Supplementary Materials

The following supporting information can be downloaded at https://www.mdpi.com/article/10.3390/plants12132409/s1, Table S1: Plant functional traits used for statistical analyses, their criteria, and data sources [61]; Table S2: Taxa of vascular plants and the entire number of propagules registered in the Traisen River during the two sampling periods (2014 and 2017).
