# Supplementary material for: Hydrochorous Seed Transport in the Lower Traisen River before and after Riverbed Restoration
Source: Plants (Basel). 2023 Jun 22;12(13):2409. doi: 10.3390/plants12132409 (PMC10347095; doi:10.3390/plants12132409)
Supplement: Supplementary file 1 [file plants-12-02409-s001.zip › plants-2418181-supplementary.pdf]

Supplementary Materials

Table S1. Plant functional traits used for statistical analyses, their criteria and data sources.

Species trait	Description	Source
Dispersal mode	Categorical. Differentiation between adaptation to anemochory (subdivided in plumed, winged and dust diaspores), hydrochory, myrmecochory, further epizochory (mammals), endozoochory. Multiple classification per species possible	[46], own observations
Seed weight	numeric, if different values from different sources - mean value	[**Error! Reference source not found.**5, 46, 47, 49, 56]
Seed size (length, width, thickness)	numeric, if different values from different sources - mean value. Regarded size of dispersed units	[46, 47], own measurements
Seed buoyancy	numeric (buoyant days) if different values from different sources - mean value	[13, 29, 51, 57, 58, 59], own measurements
Average stem height (seed release hight)		[46, 60]
Reproductive strategy	Categorized in 4 classes: s (only by seeds), ssv (mainly by seeds), sv (both seeds and vegetative) and vvs (mainly vegetative)	[46]
Life span	Categorized in annuals, monocarpic perennials and polycarpic perennials	[46]
Life forms after Raunkiær	We only categorized Phanerophytes (incl. Nanophanerophytes and chamaephytes), Hemicryptophytes, Cryptophytes (geophytes and hydrophytes taken together) and Therophytes	[46, 61]
Strategies	CSR-strategy concept after Grime	[46, 49]
Ellenberg values for moisture, nitrogen and light		[46, 50]
Eco-sociological groups	Categorized in: coniferous and deciduous forests outside the floodplain (LNW), floodplain forests and alder carrs (BAW), small sedge communities of nutrient poor mires (SC), wet meadows (MO), agricultural grasslands (MA), reeds and tall herb fen communities (PH), communities of perennial ruderals and edges (AR), pioneer vegetation of mudbanks (BI), annual ruderals and arable weeds (SO), aquatic macrophytes (H).	[46, 52], own experiences

Table S2. Taxa of vascular plants and the entire number of propagules registered in the Traisen-river during the two sampling periods (2014 and 2017). Species considered to be fully or partly hydrochorous are marked bold.

Species	short	2014 Seeds		2014 Seedlings		2017 Seedlings	
		upstream	downstream	upstream	downstream	upstream	downstream
Acer campestre L.	AceCam	1	7	1			
Acer negundo L.	AceNeg	1	11	1		46	3
Acer platanoides L.	AcePla		3				
Acer pseudoplatanus L.	AcePse		8				
Achillea millefolium L.	AchMil						1
Ailanthus altissima (Mill.) Swingle	AilAlt	2	3	1	1		
Ajuga reptans L.	AjuRep		4				
Alchemilla vulgaris L.	AlchVu		1	1			1
**_Alisma plantago-aquatica_ L. s. str.**	Alisma		8		1		
Alliaria petiolata (M. Bieb.) Cavara & Grande	AllPet		2				
**_Alnus glutinosa_ (L.) J. Gaertn.**	Alnus	1487	1893	250	225	43	2
**_Alopecurus geniculatus_ L.**	AloGen					2	
**_Alopecurus pratensis_ L.**	AloPra					1	3
**_Amaranthus_ spec.**	Amarant		8				
**_Angelica sylvestris_ L.**	AngSyl		2		1		
Arenaria serpyllifolia L. s. l.	AreSer		5		1		
Arrhenatherum elatius (L.) P. Beauv. ex J. Presl & C. Presl	ArrEla		8				
Artemisia vulgaris L.	ArtVul		12	2	7	2	
Atriplex spec.	Atriplex		4				
Ballota nigra L.	BalNig		8				
Barbarea vulgaris agg.	BarVul	1	69		8	4	1
Betula cf. *pubescens* Ehrh. s. l.	Betula	155	1094	12	14	27	10
Bidens frondosa L.	BidFro					9	13
Bidens tripartita L.	BidTri				2		1
Brachypodium sylvaticum (Huds.) P. Beauv.	BraSyl		2		5		
Bunias orientalis L.	BunOri	1	5				
Butomus umbellatus L.	ButUmb		10				
Calamagrostis epigejos (L.) Roth	CalEpi	1	259			22	6
**_Callitriche palustris_ agg.**	CalliPal					1	2
Capsella bursa-pastoris (L.) Med.	CapBur		2				
Carpinus betulus L.	CarpBet		6				
**_Chenopodium album_ agg.**	CheAlb	2	81	1	3	1	5
**_Chenopodium ficifolium_ Sm.**	CheFic	2	139		10	1	11
**_Chenopodium polyspermum_ L.**	ChePol	3	31				3
Cirsium arvense (L.) Scop.	CirsArv	14	2			1	29
Cirsium oleraceum (L.) Scop.	CirsOle						1
Clematis vitalba L.	ClemVit	59	32	35	6	24	21
Clinopodium vulgare L.	CliVul		2				
Convolvulus arvensis L.	ConArv					2	3
**_Conyza canadensis_ L.**	ConyCan	4	17	10	6	26	59
Cornus sanguinea L.	CorSan	35	34				

Table S2. Continued.

Species	short	2014 Seeds		2014 Seedlings		2017 Seedlings	
		upstream	downstream	upstream	downstream	upstream	downstream
Crataegus spec.	Crataeg	4					
Carex paniculata **L.**	CxPanicul	2	11				
Carex pseudocyperus **L.**	CxPseu	8	12	3	10	59	297
Carex remota **L.**	CxRem	2	10	6	16	19	26
Carex spec.	CxSpec.					17	34
Cyperus fuscus **L.**	CypFus		19	182	64	93	259
Dactylis glomerata L. s. str.	DacGlo					2	9
Daucus carota L.	DauCar		1		1		
Deschampsia cespitosa **(L.) P. Beauv. s. str.**	DesCes		3	6	1		1
Digitaria sanguinalis (L.) Scop.	DigSan		2				1
Echinochloa crus-galli **(L.) P. Beauv.**	EchCru	16	43		13	3	46
Elymus caninus (L.) L.	ElyCan		6				
Elymus repens (L.) Gould s. str.	ElyRep		13				
Epilobium hirsutum L.	EpiHir					1	1
Epilobium spec.	Epil_sp		15	18	14	25	71
Epilobium parviflorum Schreb.	EpiPar					3	9
Erigeron annuus (L.) Pers.	ErigAn					3	18
Euonymus verrucosus Scop.	EuoVer		3				
Eupatorium cannabinum **L.**	EupCan	5	10	29	9	548	175
Fagus sylvatica L.	FagSyl		4				
Fallopia convolvulus **(L.) Å. Löve**	Fallopia		14			4	1
Festuca pratensis **Huds. s. l.**	FesPra	1	5				
Festuca rubra **L.**	FesRub	3	5				
Filipendula ulmaria **(L.) Maxim.**	FilUlm	2	19	3	23	34	5
Frangula alnus Mill.	FraAln		2				
Fraxinus excelsior L.	FraxEx		7				
Galium aparine **L.**	GalApa					2	
Galium palustre **L. s. str.**	GalPal	1	24			2	3
Geranium robertianum L. s. str.	GerRob					1	
Helianthus annuus L.	HeliAnn		2				
Helianthus tuberosus L.	HeliTub		2				
Herniaria spec.	Herniar	1	33				
Humulus lupulus L.	HumLup	68	119	67	92	752	18
Hypericum perforatum L.	HyperPer		3		1		
Hypericum tetrapterum Fr.	HyperTet					1	
Impatiens glandulifera Royle	ImpGla	2	22		2	8	1
Impatiens parviflora DC.	ImpPar		3				
Iris pseudacorus **L.**	IriPseu	1			3	5	5
Juncus articulatus **L.**	JunArt					11	92
Juncus effusus **L.**	JunEff					7	9
Lactuca serriola **L.**	LacSer					1	1
Lolium perenne **L.**	LolPer					20	18
Lotus corniculatus agg.	LotCor		2				
Lycopus europaeus **L.**	LycEur	26	166	31	62	157	166

Table S2. Continued.

Species	short	2014 Seeds		2014 Seedlings		2017 Seedlings	
		upstream	downstream	upstream	downstream	upstream	downstream
***Lysimachia vulgaris* L.**	LysVul			1	1		
***Lythrum salicaria* L.**	LytSal			1	9	60	348
Matricaria discoidea DC.	Matric			4	3	2	13
***Mentha aquatica* L.**	MentAq	4	84	21	31	214	89
***Mentha longifolia* L. (Huds.)**	MentLon					38	15
***Myosotis* spec.**	Myosot		2				
Nasturtium officinale R. Br.	Nasturt						2
Oenothera biennis L. s.l.	OenBi					6	4
Oxalis stricta L.	OxaStr						2
Papaver spec.	Papav		17				
***Persicaria amphibia* (L.) Delarbre**	PerAmp	1	8				
***Persicaria dubia* (Stein) Fourr.**	PersDub	2	34	4	8	8	
***Persicaria hydropiper* (L.) Delarbre**	PersHyd	1	37				1
Persicaria lapathifolia* vel. *Maculosa	PersLap	4	140			990	355
***Persicaria minor* (Huds.) Opiz**	PersMin	1	10				
***Persicaria* spec.**	PersSpec.					1186	20
Petrorhagia 4axifrage (L.) Link	PetSax	1	2				
***Phalaris arundinacea* L.**	PhaAru	8	194	7	21	129	31
Phleum pratense L. s. str.	PhlPra						1
***Phragmites australis* (Cav.) Trin. Ex Steud.**	PhrAus	2	75				
Physalis alkekengi L.	PhyAlk		4				
Picea abies (L.) H. Karst.	PicAbi		5				
Plantago major subsp. *Major* L.	PlaMaj				12	19	4
Plantago media L.	PlaMed					4	3
***Poa annua* L.**	PoaAnn						1
Poa nemoralis L.	PoaNem			2			
Poa spec.	PoaSpec.				2		
***Poa trivialis* agg.**	PoaTri		9	1	31	154	19
***Polygonum aviculare* agg.**	PolyAv		8				
Potentilla supina L.	PotSup						2
Prunus avium (L.) L.	PruAvi		4				
Prunus cerasus L.	PruCer		3				
Prunus padus L.	PruPa		8				
***Ranunculus aquatilis* L.**	RanAqua						4
***Ranunculus repens* L.**	RanRep		5		1	1	
***Ranunculus sceleratus* L.**	RanSce	1	12	6	4	23	102
Reseda lutea L.	ResLut		5				
Rhamnus cathartica L.	RhaCat		5				
Robinia pseudoacacia L.	RobPse	2	10				1
***Rorippa palustris* (L.) Besser**	RorPal	1	33	3	23	112	849
Rubus spec.	Rubus		22		6		
***Rumex acetosa* L., nom. Cons. Prop.**	RumAce		2			244	15
***Rumex hydrolapathum* Huds.**	RumHyd	2	4			14	26
***Rumex obtusifolius* L.**	RumObt	11	103	16	26	96	51

Table S2. Continued.

Species	short	2014 Seeds		2014 Seedlings		2017 Seedlings	
		upstream	downstream	upstream	downstream	upstream	downstream
Rumex spec.	RumSpec.					46	22
Salix spec.	Salix					2	
Sambucus nigra L.	SamNig	13	141			1	
Saponaria officinalis L.	SapOff	1	10			26	
***Scirpus sylvaticus* L.**	SciSyl		13				
Scrophularia nodosa L.	ScrNod			1	8	2	1
***Scrophularia umbrosa* L.**	ScrUmb			1	24	7	4
***Scutellaria galericulata* L.**	ScuGal	4	15	5	2	110	318
Senecio vulgaris L.	SenVul						4
***Setaria pumila* (Poir.) Roem. & Schult.**	SetPum		12	1	2	2	9
Setaria viridis (L.) P. Beauv.	SetVir		20				
Silene dioica (L.) Clairv.	SilDio		2				
***Silene vulgaris* (Moench) Garcke s. l.**	SilVul		8			1	
Solidago gigantea vel. *canadensis*	SoliGig	6	2	22	30	350	7196
Solanum dulcamara vel. *nigrum*	SolDul		5				
Sonchus asper (L.) Hill	SonAsp	1	6			2	17
***Sparganium* spec.**	Spargan		5				1
Stachys* cf. *palustris	Stachys	1	10				
Stellaria alsine Grimm	StellAls		12				
***Stellaria aquatica* (L.) Scop.**	StellAqu		72		3	3	
Stellaria spec.	StellSpec.		3				
Stellaria media agg.	StellMed		15		1	34	2
Tanacetum vulgare L.	TanVul					2	6
***Taraxacum officinale* agg.**	Taraxac		5	15	17	63	56
***Thalictrum flavum* L.**	ThaFla					2	
Thuja occidentalis L.	ThujaO	5	27	1	3		
Tilia cordata Mill.	TilCor	2	5				
Tilia platyphyllos Scop.	TilPla	3	5				
***Tussilago farfara* L.**	TusFar						1
***Typha* spec.**	Typha	1	1				
***Urtica dioica* L.**	UrtDio	159	1684	272	255	9513	233
Valerianella locusta (L.) Laterr.	ValLoc					1	
Valeriana officinalis agg.	ValOff		1	7	7	1	1
***Veronica anagallis-aquatica* L.**	VerAna			2	3	10	3
Verbascum densiflorum Bertol.	VerDen						1
***Veronica hederifolia* agg.**	VerHede					1	
***Zannichellia palustris* L.**	ZanPal		3				
